# A cell-centered, agent-based framework that enables flexible environment granularities

**DOI:** 10.1186/s12976-016-0030-9

**Published:** 2016-02-02

**Authors:** Ryan C Kennedy, Glen EP Ropella, C Anthony Hunt

**Affiliations:** Department of Bioengineering and Therapeutic Sciences, University of California, San Francisco, CA USA; Tempus Dictum, Inc., Milwaukie, OR USA

**Keywords:** Biomimetic, Cell behavior, Off-Lattice, Delaunay, Voronoi, Morphogenesis, Mechanistic explanations, Modeling

## Abstract

**Background:**

Mechanistic explanations of cell-level phenomena typically adopt an observer perspective. Explanations developed from a cell’s perspective may offer new insights. Agent-based models lend themselves to model from an individual perspective, and existing agent-based models generally utilize a regular lattice-based environment. A framework which utilizes a cell’s perspective in an off-lattice environment could improve the overall understanding of biological phenomena.

**Results:**

We present an agent-based, discrete event framework, with a demonstrative focus on biomimetic agents. The framework was first developed in 2-dimensions and then extended, with a subset of behaviors, to 3-dimensions. The framework is expected to facilitate studies of more complex biological phenomena through exploitation of a dynamic Delaunay and Voronoi off-lattice environment. We used the framework to model biological cells and to specifically demonstrate basic biological cell behaviors in two- and three-dimensional space. Potential use cases are highlighted, suggesting the utility of the framework in various scenarios.

**Conclusions:**

The framework presented in this manuscript expands on existing cell- and agent-centered methods by offering a new perspective in an off-lattice environment. As the demand for biomimetic models grows, the demand for new methods, such as the presented Delaunay and Voronoi framework, is expected to increase.

**Electronic supplementary material:**

The online version of this article (doi:10.1186/s12976-016-0030-9) contains supplementary material, which is available to authorized users.

## Introduction

There is a growing need for new methods to cater to increasingly complex biological models, which aim to provide better mechanistic explanations of biological phenomena. We describe and make available an early stage simulation framework that enables a dynamic Delaunay and Voronoi (D/V) off-lattice environment to be created and used by biomimetic agents. This framework can accommodate a variety of uses, among which are those that adopt a cell- or entity-centered perspective. It is intended to expand the repertoire available to modelers by helping to make irregular grids part of their standard toolkit.

## Background

Three scientifically important needs provided motivation for this simulation framework. When one is engaged in improving mechanistic explanations of cell level phenomena, we aim to make it easier to: 1) modify mechanistic granularity within a simulation where and when that is needed to improve explanatory insight [1]; 2) alter the focus or perspective of a simulation in much the same way wet-lab biologists adjust the focus of their experiments; 3) acknowledge, identify, represent, and begin explaining multilevel uncertainties within and between comparable observations made using different wet-lab systems. An important biological focus for us has been improving mechanistic explanations of particular phenomena at cell and multicell levels, primarily in vitro, during such fundamental processes as wound healing, formation and maintenance of a single central lumen during early cystogenesis [2], and multicellular collective invasion that is characteristic of carcinomas [3, 4].

Research presented in the cited papers employ methods representative of the field. The focus of experiments necessarily shifts from one aspect to another as phenomena change and evolve. For example, early in cystogenesis [2], attention may focus on pre-luminal events occurring at the apical interface of two or more cells. Multiple visualization methods are employed; examples include differentially staining particular proteins or using cells capable of expressing fluorescent versions of particular proteins. Events elsewhere in the multicell structures are deemed less critical, and thus may not be measured or observed. Later in the cystogenesis process, attention may shift to characteristics of whole cysts; for example, fine grain details at cell-cell interfaces may be deemed less influential to evolving system-level phenotype. Fluorescent staining of cell nuclei may enable measuring the relative arrangement of cells in a cyst, yet data identifying locations of cell boundaries and/or cell-cell interfaces may not be available because they were not visualized or measured. When studying cell behavior in tumor organoids [4], an experiment’s scale of focus may shift between numbers of invasive multicell leader structures emanating from particular tumor organoids to behavior of individual leader cells within a single leader structure.

The preceding examples illustrate that in such experiments there is always uncertainty about aspects and features not measured. Ideally, we would like to acknowledge, even represent, such uncertainty within our simulation models by avoiding over-committing to the simulation of particular details when and where there is little or no data against which to validate those commitments. However, doing so is challenging [1] and even problematic if one begins a modeling and simulation project by specifying in advance how and at what level of detail a mechanism’s spaces, entities, and activities will be represented. Those commitments begin when the modeler follows dominant practices and, for example, simply chooses in advance to use a regular grid. We envision the D/V grid providing the capability to fine or coarsen a model’s local scale of focus. An irregular grid allows us to selectively fine or coarsen the scale in one region more or less than in other regions, thereby adjusting granularity.

Explanations of, and theories about, cell level and multicell phenomena, such as those cited above, are offered based on inferences drawn from observing many images, yet only a few images, thought by the said research authors to be typical or representative, are included in publications. A natural inclination for a modeler is to focus first on the images provided, begin creating computational descriptions of those images, and then hypothesize and describe possible event sequences to fill the gaps separating the created depictions. So doing is made straightforward when one commits upfront to using a regular grid. Once that commitment is made, the modeler is at risk of restricting attention to those mechanistic entities and activities that are easily instantiated using the selected grid.

Early commitment to a regular grid also supports favoring the observer’s perspective rather than the biology’s perspective. Longer term, we anticipate that “decisions” made by each cell will contribute to a greater phenomenon based only on information available in its local environment—the cell’s perspective—and the resolution or granularity with which the cell samples or measures those features, thereby enabling observation and validation from cell-and system-levels. Further, we can anticipate that location within the larger system will lead to location-dependent differences in mechanisms. We envision the D/V grid being used by an individual agent representing an individual cell (cell cluster or other entity) to fine or coarsen its local focus. Again, because our framework utilizes an irregular grid, the cell agent will be able to selectively fine or coarsen granularity at one location more or less than elsewhere.

### Agent-based modeling

We expect researchers interested in our framework will have familiarity with agent-based modeling (ABM) techniques, and we encourage those interested in further details to seek Macal and North’s tutorial [5]. Agent-based methods are adept at modeling natural phenomena, and an ABM approach helps facilitate the needs of the cell-centered framework. ABMs can represent complex entities and behaviors at an agent-level; each entity, or agent, implements a set of rules and has its own set of properties. Numerous agent classes can exist within a model, and the interactions between agents can lead to global, or emergent, properties. Because mechanisms in ABM are often informally specified, with few or no theoretical constraints, natural phenomena can be simulated more directly, in contrast to those methods that require arching principles which may bias simulation results. ABM has been applied to a wide range of disciplines, from social networking to biology. D/V grids are a natural fit with ABM, as D/V grids facilitate the emergence of a spatial structure rather than one that is based upon prior assumptions.

### Lattice and off-lattice spatial representations

Spatial representations in cell modeling can be categorized as lattice (discrete) or off-lattice (continuous); we focus the framework on an off-lattice spatial representation, D/V grids, which give us the capability to utilize neighborhood-based information at an individual agent’s level.

Lattice-based models limit cells to defined spatial boundaries or grids and are most frequently used within the cellular Potts model (CPM). The CPM is an extensible technique to simulate a variety of cell behaviors, such as morphogenesis, sorting, adhesion, as well as tissue simulations and virtual tumors [6–14]. In the CPM, cells exist within a lattice and are connected with bonds represented by various equations; cell behaviors are governed largely by “energies.” They have the ability to model cells in a variety of shapes and sizes and can utilize a number of grid representations. Conventional agent-based models utilize lattices and the correlating neighborhoods for most agent-to-agent interactions. Hexagonal grids are increasingly used [15] and have some inherent advantages.

**Fig. 1:**
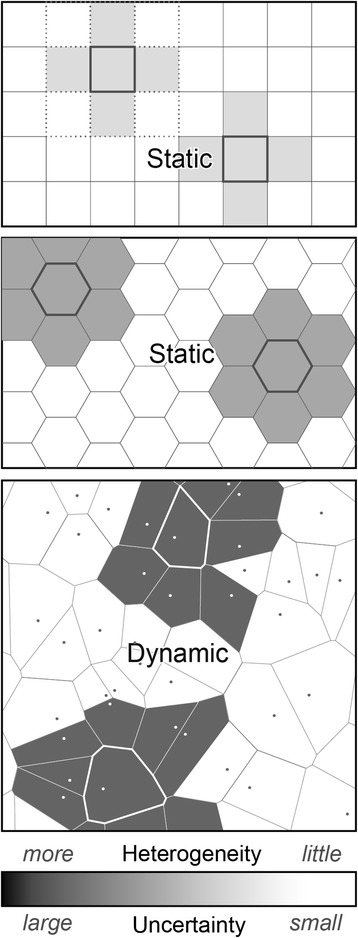
Comparison of Grid Types. Shown are three fundamental grid types: square, hexagonal, and Voronoi. The Voronoi grid’s dynamic spatial structure is generated and updated at run time. A 1-hop von Neumann neighborhood from the marked cell is shaded according to the spectrum at the bottom. For the upper left cell, the eight-cell Moore neighborhood is also identified. Each particular modeling and simulation task can be characterized by locations on the spectrum at the bottom that respond to these two use cases. Select a location range that characterizes 1) uncertainty about mechanisms that may explain generation of the targeted biological phenomenon; and 2) heterogeneity of targeted wet-lab measurements. Use of a uniform, static square or hexagonal grid requires accumulating two categories of assumptions: 1) those needed to make spatial uncertainties compatible with grid granularity, and 2) those needed to map simulated to wet-lab phenomena. The flexibility of Voronoi grid structures can be extended to represent uncertainty. It can be focused differently in different spatial regions and/or as an execution progresses. Consequently, fewer assumptions may be needed

When dynamic spatial structures are needed, an off-lattice representation is useful. In an off-lattice spatial representation, continuous values are used to represent spatial boundaries, such as the extent of a cell. Vertex, force-based, and the subcellular element method are examples of off-lattice model representations. Vertex models depict cells as polygons, and have been used in models of cell mechanics and cell sorting [16–18]. Force-based models have a number of representations, such as ellipsoids, which are used in Palsson’s model of cell movement [19]. The subcellular element method, which has been used in studies of cell mechanics, represents a cell as a number of subcellular elements, which are governed by equations of motion [20]. Galle et al. [21] utilized this method in their off-lattice model of carcinoma growth. Off-lattice models are typically more computationally expensive than lattice-based models, but there has been recent effort to offset computation to the graphics processing unit [22]. Extra computation is needed to determine cell neighbors, and the spatial representation must be continuously updated. There is an inherent need for off-lattice capabilities in cell modeling, particularly when the cells are motile. Off-lattice models can come very close to approximating the shape and pattern of biological cells [23], and that can make the representation more biologically realistic [24–31]. Observations of protrusions within the epithelium [3] highlight a specific case where accurately simulating said behavior would be difficult in a lattice environment. Moreover, in morphogenesis, the benefits of an off-lattice grid are evident. D/V grids provide a means to explore a number of use cases. Several are described in subsequent sections. In a Voronoi tessellation of a given set of center points in a plane, every point in a given area is closer to the center point in its area than to any other center point. We refer to this area as a Voronoi cell, hereafter V-cell. The dual graph of the Voronoi tessellation is the Delaunay triangulation; the same set of points can be triangulated in a manner such that there is no point inside the circumcircle of any triangle. Bowyer and Watson concurrently described algorithms for construction of Delaunay triangulations [32, 33]. Advantages of D/V grids include a more realistic neighborhood for cells in which we can abstract from a traditional, regular (scaled) grid and form dynamic cells of varying or uncertain shapes and sizes, with varying numbers of neighbors, capturing some prespecified level of detail and complexity (granularity). A comparison of aforementioned grid types is presented in Fig. 1.

There has been only limited work incorporating off-lattice D/V grids into ABM methods. Most notably, the Meyer-Hermann group has developed dynamic force-based models that utilize D/V grids [34], which have been used in studies of multicellular tumor spheroids [35, 36]. Drasdo et al. [37] built upon the models from the Meyer-Hermann group to model three-dimensional (3D) tumor growth. An additional example of off-lattice modeling is provided by Dillon et al. [38] in their model of cell growth and division. Pathmanathan et al. [39] describe tissue models that utilize D/V grids, but not in a direct ABM syntax. Honda used D/V grids to represent the wing epidermis in butterflies [40]. The Chaste software library [41] supports models with D/V grids, but largely focuses on cardiac modeling. Virtual Cell [42] provides a framework for off-lattice representations and provides mathematical solvers but without an agent-based core.

D/V grids are most appropriate for modeling use cases where the spatial medium (or organization) is part of the experimental target, an aspect of the model to be studied, as opposed to an assumption programmed by the developer. Similar modeling objectives are sought by network-based models where the organization and interactions of model components arise as a consequence of the model. It is in this manner that we aim to differentiate our framework from existing approaches.

## Framework and Methods

The framework is implemented in MASON [43] with heavy use of the Visualization Toolkit (VTK) [44]; it is available at simtk.org [45]. MASON is a powerful Java-based simulation framework, and notable advantages of MASON include speed, a distinct separation of display objects from the core simulation, and the built-in ability to checkpoint simulations [46]. VTK is an advanced system for image processing and 3D graphics. As an open source toolkit, VTK has been incorporated into many systems, has a rich set of algorithms, and has been extensively used for medical imaging. While it is written in C++, wrappers exist that make it accessible to other programming languages. VTK supports Delaunay triangulations, both in two-dimensional (2D) and 3D space, but it does not have built in mechanisms to compute Voronoi tessellations. With MASON, we utilize (Java) VTK and build upon its capabilities to allow for Voronoi tessellations. MASON and VTK allow for the 2- and 3D display of data.

### 2-Dimensional V-cells

**Fig. 2:**
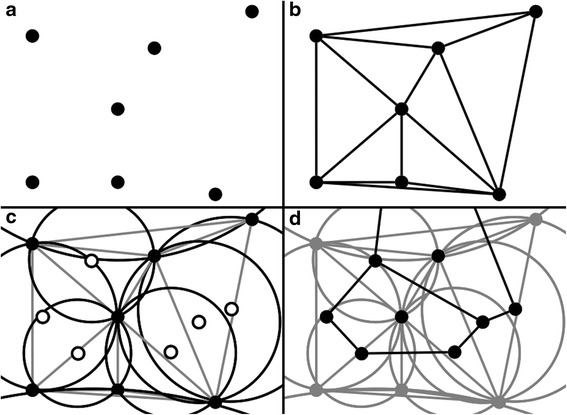
V-cell Calculation Process. The figure above shows the process by which we calculated the extents of V-cell agents in 2D. A set of points are shown in panel (**a**), and a triangulation of those points are shown in panel (**b**). For each of the 8 triangles in (**b**), a circumcircle and its center point, shown as a hollow point, is drawn, and shown in panel (**c**). Panel (**d**) shows the connected center points and the corresponding 2 V-cells

Development of the framework progressed incrementally: 1) A set of randomly placed 2D points was implemented as MASON agents; 2) We triangulated the points with VTK and displayed the results, in part for face verification; 3) We added movement and time. Agents were allowed to randomly move about, and the triangulation was recalculated at each time step. From an agent’s perspective, methods were developed to obtain neighboring agents; 4) The framework calculated the extent of a given V-cell, starting by utilizing VTK’s circumcircle method on each triangle. The structure of the system was represented similarly to that of the table in Bowyer [32]. Calculating the table was an involved process, as VTK does not have the built-in capability to calculate Voronoi tessellations. Where possible, we utilized VTK’s built-in methods to perform mathematical calculations; 5) Along the way, we added the capability to load a simulation from an input file. The input file contains the continuous coordinates of V-cell center points, as well as the V-cell type and information, enabling different V-cell types to respond differently to external stimuli. Together with this input, a user can now create and simulate various behaviors. Figure 2 and the algorithm in Additional file 1 further detail this process.

### 3-Dimensional V-cells

**Fig. 3:**
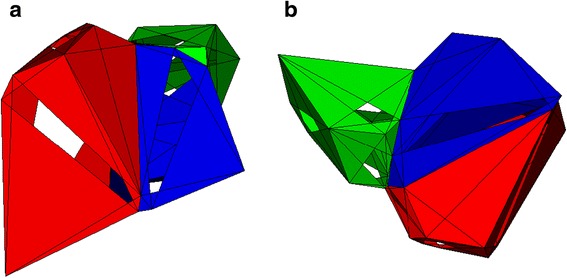
Three-dimensional V-cells. This figure shows three V-cells (for simplicity) in a 3D space. Here, V-cells are displayed with incomplete faces to facilitate viewing. Shared faces can be seen between the V-cells. Different V-cell colors represent different V-cell types. We show the same three V-cells from different perspectives for clarity, panels (**a**) and (**b**)

**Fig. 4:**
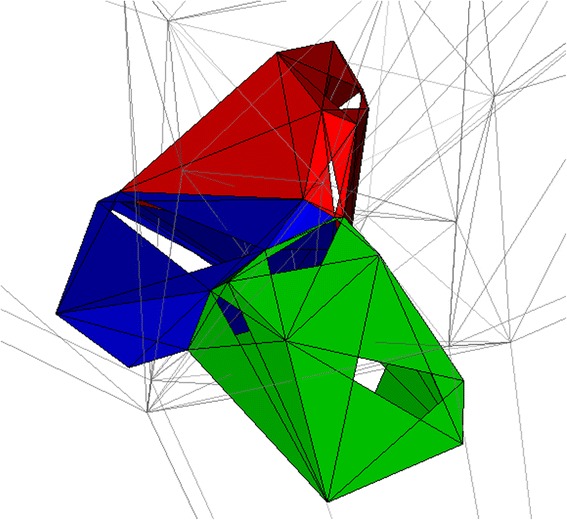
Three-dimensional V-cells with Triangulation. The cells from Figure 2 are shown in a different view with their underlying triangulation, which can be inferred as media. The media must completely encompass all V-cells to create the encapsulated system, and the full extents of the media are not shown

Adding 3D space was more complex. Generating a virtual table as we did for 2D space presented many computational issues, and we instead relied on computational geometry techniques. VTK has the ability to triangulate a set of 3D points, but, again, it cannot calculate a Voronoi tessellation. Triangulating a set of 3D points generates a set of tetrahedra, each corresponding to a single V-cell. To calculate the faces of a given V-cell, we first determine the two tetrahedra that share the center point with that V-cell, as well as the shared faces. For each of these two adjacent tetrahedra, we use the VTK algorithm to calculate the circumspheres. Combined with the circumsphere of the given V-cell, we can generate a triangle. Repeated applications of this approach generate a set of triangles that make up the surface of a given V-cell face. One could think of these surfaces as cell-environment interfaces. The algorithm featured in Additional file 2 further details this process. Figure 3 shows sample 3D V-cells and Fig. 4 shows sample 3D V-cells with the underlying triangulation visible.

## Results

Improving explanatory insight into the generation of biological phenomena, for example those observed during wound healing and morphogenesis, is critical to advancing biomedical science. Cell-centered models can represent the behavior of systems of individual interacting cells and tissue functional units to study their collective behaviors. While tracking the behavior of an individual cell during a wet-lab experiment can at best be challenging, system-level measurements are routine. We engineered the framework to facilitate study of biological cells and have demonstrated use by modeling basic cell behavior, from a cell’s perspective, utilizing 2D and 3D D/V grids as a basis for V-cells.

### V-cell representation

The framework can support an arbitrary number of V-cell agent types, representing many entities. Each V-cell type is recognized by specific behaviors in the method and operates from a V-cell-based perspective. V-cells agents rely on their surrounding neighborhoods to make decisions. Partly because V-cell agents are not on a grid, decisions are more isolatable from the surrounding environment. The use of a D/V grid means that agents have no regular neighborhood shape. Instead, local computations are made for an individual V-cell. Neighbors are considered as V-cells that are a single hop away from the observing V-cell, meaning other V-cell centers to which the V-cell center point is directly connected (there exists a edge between the V-cells). Since V-cell center points make up the nodes for the triangulation, neighbors can be identified (calculated) from the triangulation. When a V-cell agent moves, its center point moves and the triangulation must be recalculated. The resulting V-cells are calculated and change location and shape automatically depending on the agent’s rules and agenda; many V-cells are impacted when a single V-cell moves. Similarly, when a given V-cell grows or shrinks, surrounding V-cells may be pushed or pulled.

#### Use case: biological cells

**Fig. 5:**
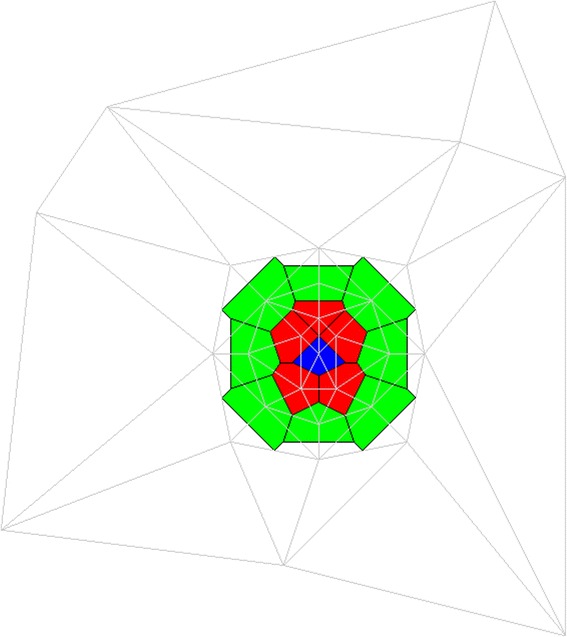
Two-dimensional V-cells. Three types of V-cells are shown in a 2D space. A single blue lumen cell is surrounded by lec (*red*) and mec (*green*) cells. Again, the underlying triangulation is shown. While the cells shown here occupy a somewhat regular shape, this does not need to be the case

The biological referents for demonstrations are simple cell culture cysts having an inner layer of luminal epithelial cells enclosing a lumen and an outer layer of myoepithelial cells. Discussion of these cell types and structures is beyond the scope of this paper and can be found elsewhere [47, 48]. Figure 5 is an illustration of an idealized 2D cyst showing the basic layout for 2D V-cells, and Additional file 3 provides a video of cysts which shows movement and rotation [49]. Demonstrations employ three components. A lmn agent maps to a cyst lumen. Lec and mec agents map to luminal epithelial cells and myoepithelial cells, respectively.

Agent behaviors are governed by axioms developed by domain experts. We have implemented mechanisms to mimic movement, growth, division, and death in 2D and 3D space. Lec and mec can move randomly or in a general direction, based on the underlying Cartesian space. For growth, the agent increases its area or volume, while pushing out neighboring agents. In division, the agent creates a copy of itself. The copy, or daughter agent, inherits traits from its parent. Additionally, agents can be removed from the system. Removal can map to a cell disintegration following death. When an agent is removed, surrounding agents can compete to fill the void. V-cell absorption is also demonstrated; specifically, a mec cell is shown to be absorbed by a cyst. We have implemented an additional behavior in 2D: V-cell rotation, in which a set of agents rotates around another agent. We demonstrate lec agents rotating around a lmn agent in Additional file 4. It is important to note that we have not attempted to implement a fully falsifiable hypothesis for an actual referent. Rather, we have developed a framework whereby the individual agents make decisions rather than utilizing observer requirements, and we have specified several examples through the form of basic use cases. We do not, at this stage, believe it necessary to have a fully falsifiable referent to demonstrate how this method could be used to glean new explanatory insights from an agent’s perspective.

Additional files 4, 5, 6, 7, 8, 9 and 10 are illustrative videos depicting the aforementioned behaviors. Additional file 4 demonstrates 2D agent rotation. Additional file 5 shows 2D agent movement, division, and death, while Additional file 6 demonstrates 2D agent growth. Additional file 7 shows 2D absorption. We visualize 3D agent movement, division, and death in Additional file 8; we show 3D agent growth in Additional file 9. Additional file 10 shows absorption in 3D. Finally, Additional file 11 shows the underlying tetrahedral mesh similar to what is used in Additional file 10. The speed of each video is decreased for clarity.

## Discussion

We provide a new framework for use within the realm of cell- or entity-centered modeling. Although we demonstrate only a few basic cell behaviors, the framework is designed to be extensible to accommodate a variety of use cases. Examples include aspects of morphogenesis and tissue repair in vitro and in vivo. Objects that map to particular mobile molecules, including one or more drugs, can be added. Doing so is expected to facilitate using simulations to explore competing explanations of disease progression in the presence and absence of a therapeutic intervention.

Networks naturally lend themselves to study via D/V grids. Nodes can map to V-cell centers, with the range of “awareness” or capacity denoted by the size of the V-cell. Agents at nodes can communicate with other agents at other nodes based on the number of nodes between them. In this manner, irregular grid structures can be used to model, for example, neuronal tissue, where mobile objects representing transmitters can traverse the graph.

The framework’s design enables study of interfacial regions between neighboring entities. In the demonstrations, an interface between agents need not have a specific biological counterpart. Rather, it can be mapped to cell-cell and/or cell-environment interfaces. Dynamic interfaces between agents during simulations are necessarily precise. The mapping to biology, however, should include appropriate uncertainties.

## Conclusions

We demonstrate and make available a new framework that expands upon traditional cell- and agent-centered methods. Our framework provides opportunities for several use cases. The methods can be applied across domains as a framework. V-cells can be used to represent any entity, at various granularities. We foresee these methods facilitating new ways to think about and better explain biological phenomena.

## Availability and Requirements

Our framework is available as source code, and we also include the VTK Java bindings and MASON Java archive (JAR) files in the download. Installation instructions on Ubuntu are included as documentation within the download.

**Project Name**: DVFramework

**Project home page**: https://simtk.org/home/dvframework

**Operating system**: Platform independent

**Development Environment**: Java 1.6

**Other requirements**: MASON 15, VTK 5.8

**License**: GNU General Public License v3

**Any restrictions to use by non-academics**: none

## Additional files

Additional file 1:
**2D V-cell Algorithm.** Algorithm 1 provides pseudocode for the process by which we calculated V-cell extents in 2D. (PDF 17 kb) Additional file 2:
**3D V-cell Algorithm.** Algorithm 2 provides pseudocode for the process by which we calculated V-cell extents in 3D. (PDF 17 kb) Additional file 3:
**Alveolar-like cyst morphogenesis.** This video shows alveolar-like cysts which rotate and move about in Matrigel. In particular, a cyst can be seen rotating in the lower left quadrant beginning about 27 seconds in, and continuing to rotate for about 8 seconds. Moving cysts are abundant throughout the video. (MOV 4779 kb) Additional file 4:
**2D Cell Rotation.** This video simulates rotation of lec about the lmn. Lumen and mec cells do not move, but are impacted by the moving lec. (MOV 4997 kb) Additional file 5:
**2D Cell Movement, Division, and Death.** In this video, we start with multiple V-cells that move about randomly. The triangulation is calculated at each time step and, notably, flipping of edges in the triangulation does not impact V-cells. After 10 seconds, a new lmn V-cell is added. After an additional 9 seconds, the new lmn V-cell is removed. (MOV 5535 kb) Additional file 6:
**2D Cell Growth.** This video highlights the growth of a lmn cell and its effect on surrounding cells. After 8 seconds, the lmn V-cell begins to grow. (MOV 3021 kb) Additional file 7:
**2D Cell Absorption.** A cluster of V-cells is shown randomly moving in the center of the video. A mec V-cell is shown moving from the lower left quadrant toward the cluster of V-cells, and it is eventually absorbed by the mec in the cluster. The mec V-cell changes shape as it traverses through the media toward the cluster of V-cells. (MOV 8466 kb) Additional file 8:
**3D Cell Movement, Division, and Death.** Similar to Additional file 4, we start with multiple V-cells that randomly move. After 7 seconds, we add a new lec V-cell. After an additional 7 seconds of random movement, we remove the lec V-cell. (MOV 3821 kb) Additional file 9:
**3D Cell Growth.** This video highlights the growth of a lec cell and its effect on surrounding V-cells. 2 seconds into the video, we add the new V-cell and it continues to grow throughout the remainder of the video. The new V-cell’s impact is seen on its neighbors. (MOV 2470 kb) Additional file 10:
**3D Cell Absorption.** A set of 3 V-cells are shown in the upper right quadrant in this video, while a single mec V-cell is shown in the lower left quadrant. As the video progresses, the single mec moves toward and is eventually absorbed by the other mec V-cell in the cluster. The shape of the moving mec changes as it moves through the media. (MOV 19651 kb) Additional file 11:
**3D Tetrahedral Mesh.** An example tetrahedral mesh, which makes up the media, is shown for 3D V-cells. Close inspection will reveal the same basic initial state as that from Additional file 10, albeit from a different angle. V-cell extents are calculated based upon this mesh. (PNG 975 kb)
