# Supplementary material for: A cell-centered, agent-based framework that enables flexible environment granularities
Source: Theor Biol Med Model. 2016 Feb 2;13:4. doi: 10.1186/s12976-016-0030-9 (PMC4736144; doi:10.1186/s12976-016-0030-9)
Supplement: Additional file 1: — 2D V-cell Algorithm. Algorithm 1 provides pseudocode for the process by which we calculated V-cell extents in 2D. (PDF 17 kb) [file 12976_2016_30_MOESM1_ESM.pdf]

2D V-cell Algorithm

-Let P be the set of input points in 2D space
-DELAUNAY2D(P) returns the triangulation for a given set of points P, which represent the V-cell centers
-CIRCUMCENTER(t) returns the center of circle which passes through all vertices of the given triangle t
-GETNEXTVERTEX(v_1,v_2,t.id) takes two vertices, v_1 and v_2, and the id of the current triangle, t.id, and returns the next triangle with forming points v_1 and v_2 and which also shares an edge with t.id, without backtracking
-COUNTERCLOCKWISE(pointsList) takes a list of points and returns the points in sorted counterclockwise order
-CREATEPOLGYON(pointsList) takes a list of counterclockwise points and creates a polygon

```
procedure CALCULATEVCELLS2D(P)
T ← DELAUNAY2D(P)

for all p_i ∈ P do
  for all t_j ∈ T do
    if t_j.contains(p_i) then                    //if p_i exists as a point in t_j
      centerFormingPoint ← t_j.pointOne
      secondFormingPoint ← t_j.pointTwo
      currentTriangle ← t_j
      startTriangle ← t_j
      triangleList.add(currentTriangle)
      circumcenterPointsList.add(CIRCUMCENTER(currentTriangle))
      repeat
        t_k ← GETNEXTVERTEX(centerFormingPoint, secondFormingPoint,
          currentVertex)
        currentVertex ← t_k
        secondFormingPoint ← t_k.pointTwo
        circumcenterPointsList.add(CIRCUMCENTER(currentTriangle))
      until currentTriangle.equals(startTriangle)
      vCells.add(p_i.id)
      vCenters.add(circumcenterPointsList)
    endif
  end for
end for

for all circumcenterPointsList ∈ vCenters do
    counterClockwisePointsList ← COUNTERCLOCKWISE(circumcenterPointsList_i)
    vCellPolygonList.add(CREATEPOLGYON(counterClockwisePointsList))
    vCellCentersList.add(vCells.get(i))
  end for
end for

return vCellPolygonList, vCellCentersList
```
