# Supplementary material for: A cell-centered, agent-based framework that enables flexible environment granularities
Source: Theor Biol Med Model. 2016 Feb 2;13:4. doi: 10.1186/s12976-016-0030-9 (PMC4736144; doi:10.1186/s12976-016-0030-9)
Supplement: Additional file 2: — 3D V-cell Algorithm. Algorithm 2 provides pseudocode for the process by which we calculated V-cell extents in 3D. (PDF 17 kb) [file 12976_2016_30_MOESM2_ESM.pdf]

3D V-cell Algorithm

-Let P be the set of input points in 3D space
-DELAUNAY3D(P) returns the 3D triangulation (as a set of tetrahedra) for a given set of points P, which represent the V-cell centers
-CIRCUMCENTER(t) returns the center of sphere which passes through all vertices of the given tetrahedron t
-GETFACES(p,t) returns the 3 triangles from tetrahedron t which share a point with p
-GETADJACENTTETRA(p,tri) returns the tetrahedra which shares a point with p and a face with triangle tri
-ADDTRIANGLE(p_1,p_2,p_3) returns a triangle containing given points p_1, p_2, and p_3

```
procedure CALCULATEVCELLS3D(P)
T ← DELAUNAY3D(P)

for all p_i ∈ P do
  for all t_j ∈ T do
    if t_j.contains(p) then                      //if p_i exists as a point in t_j
      p_i.tetras.add(t_i)
    end if
  end for
end for

for all p_i ∈ P do
  for all t_j ∈ p_i do
    t_j.center ← CIRCUMCENTER(t_j)
    t_j.faces ← GETFACES(p_i,t_j)
    for all triangle_k ∈ t_j.faces do            //for the 3 triangle faces
      triangle_k.tetra ← GETADJACENTTETRA(p_i,triangle_k)
      triangle_k.tetraCenter ← CIRCUMCENTER(triangle_k.tetra)
    end for
    vCellList(p_i).ADDTRIANGLE(t_j.center,triangle_0.tetraCenter,triangle_1.tetraCenter)
    vCellList(p_i).ADDTRIANGLE(t_j.center,triangle_0.tetraCenter,triangle_2.tetraCenter)
    vCellList(p_i).ADDTRIANGLE(t_j.center,triangle_1.tetraCenter,triangle_2.tetraCenter)
  end for
end for

return vCellList
```
