# Supplementary figures and images for: A cell-centered, agent-based framework that enables flexible environment granularities
Source: Theor Biol Med Model. 2016 Feb 2;13:4. doi: 10.1186/s12976-016-0030-9 (PMC4736144; doi:10.1186/s12976-016-0030-9)

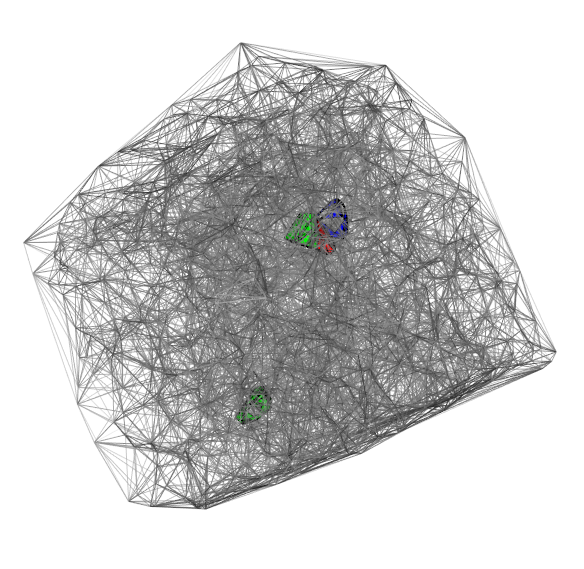

Supplement: Additional file 11: — 3D Tetrahedral Mesh. An example tetrahedral mesh, which makes up the media, is shown for 3D V-cells. Close inspection will reveal the same basic initial state as that from Additional file 10, albeit from a different angle. V-cell extents are calculated based upon this mesh. (PNG 975 kb) [file 12976_2016_30_MOESM11_ESM.png]
